# Disseminated Histoplasmosis as an AIDS-Defining Illness Presenting as Fever of Unknown Origin in an 11-Year-Old Female

**DOI:** 10.1155/2019/9417102

**Published:** 2019-05-13

**Authors:** Kathryn E. Kalata, Christina Osborne, Amy Willis, Kacey Navarro, Laura Z. Fenton, Christiana Smith

**Affiliations:** ^1^University of Colorado School of Medicine, 13001 E. 17th Place, Aurora, CO 80045, USA; ^2^Pediatric Infectious Diseases, University of Colorado School of Medicine, 13123 East 16th Avenue, Box 055, Aurora, CO 80045, USA; ^3^Pediatric Hospital Medicine, University of Colorado School of Medicine, 13123 East 16th Avenue, Box 302, Aurora, CO 80045, USA; ^4^Pediatric Infectious Diseases, Children's Hospital Colorado, 13123 East 16th Avenue, Box 055, Aurora, CO 80045, USA; ^5^Pediatric Radiology, University of Colorado School of Medicine, 13123 East 16th Avenue, Box 125, Aurora, CO 80045, USA

## Abstract

A previously healthy 11-year-old female, who emigrated from Central America four years prior, was admitted with eight days of fever, night sweats, and anorexia. Past medical history included severe bronchiolitis, varicella, and hepatitis A as a child. Upon admission, her physical exam was significant for nontender cervical lymphadenopathy, intermittent erythematous papules on the upper extremities, and mild abdominal tenderness. Initial laboratory studies revealed leukopenia, anemia, elevated inflammatory markers, and antibodies to HIV-1 in the patient's serum and cerebrospinal fluid. Computed tomography scan was remarkable for many small nodules throughout the lungs and widespread lymphadenopathy. Additional testing confirmed the diagnosis of HIV/AIDS with a CD4 count of 52 cells/mm^3^, complicated by disseminated histoplasmosis. This case is significant because it represents a late presentation of vertically transmitted HIV with disseminated histoplasmosis in a nonendemic region as the AIDS-defining illness. This highlights the importance of maintaining a broad differential for opportunistic infections, especially among those who have spent a significant amount of time in a country where unusual pathogens may be more common. This case also considers the utility of antigen testing as a sensitive diagnostic test in immunocompromised patients.

## 1. Introduction

With the advent of antiretroviral therapy (ART), improvement in HIV screening, and prevention of mother-to-child transmission programs, the number of children living with untreated HIV/AIDS or presenting with AIDS-defining opportunistic infections is relatively small, especially in well-resourced countries. While most untreated children with vertically transmitted HIV become symptomatic and often die before age 5, rarely they can present later in life [1, 2]. There are few published cases of vertically transmitted HIV/AIDS with initial presentation in adolescence. The most common presentations of AIDS among children in the United States (U.S.) include *Pneumocystis jirovecii* pneumonia, wasting syndrome, esophageal candidiasis, *Cytomegalovirus* pneumonia, and HIV encephalopathy [3]. Here, we present an unusual case of an adolescent presenting with disseminated histoplasmosis as an AIDS-defining illness in a nonendemic area.

## 2. Case Presentation

A previously healthy 11-year-old girl presented with 8 days of fever, night sweats, and subjective weight loss. Her fevers occurred every 12 hours and reached a maximum of 39.4°C. Additional symptoms included headaches, dizziness, nausea, intermittent right-sided abdominal pain, and anorexia. The patient also reported an intermittently pruritic rash on her arms. She had no respiratory symptoms, emesis, or diarrhea.

Past medical history was significant for multiple episodes of bronchiolitis requiring hospitalization before age 2, varicella with severe mucosal involvement requiring hospitalization for nasogastric feeding at age 4, and hepatitis A at age 7. Growth and neurologic development were normal. The patient was born in Central America and immigrated to the U.S. 4 years prior. Exposure history was significant for consumption of unpasteurized cow milk while in Central America. A maternal uncle had been recently diagnosed with tuberculosis, but the patient had not had contact with him for more than 4 years. There was no other significant family history.

Physical exam revealed a thin female (weight 33.1 kg, 22% for age; body mass index 15.5 kg/m^2^, 16%) with enlarged, mobile, nontender cervical lymph nodes bilaterally but no palpable axillary or inguinal lymph nodes. There were small erythematous papules on the flexor surface of her left antecubital fossa and right first metacarpophalangeal joint. She had mild abdominal tenderness most significant in the right upper quadrant, but no hepatosplenomegaly or mass.

### 2.1. Initial Diagnosis

**Figure 1:**
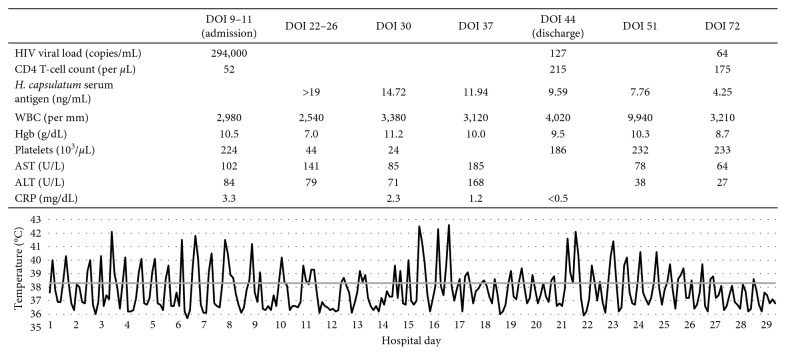
Presented laboratory values and temperature by day of illness (DOI).

Laboratory values at admission on day of illness (DOI) 9 were notable for leukopenia, anemia, and mildly elevated C-reactive protein (CRP) (Figure 1). The erythrocyte sedimentation rate was greater than 145 mm/hr. Aspartate and alanine aminotransferase were elevated. Cerebrospinal fluid (CSF) analysis showed no leukocytes and normal protein and glucose. A fourth-generation antigen-antibody test was positive for antibodies to HIV-1. HIV RNA PCR demonstrated 294,000 copies/mL in peripheral blood and 504 copies/mL in CSF; initial CD4 T-lymphocyte count was 52 cells/mm^3^.

**Figure 2:**
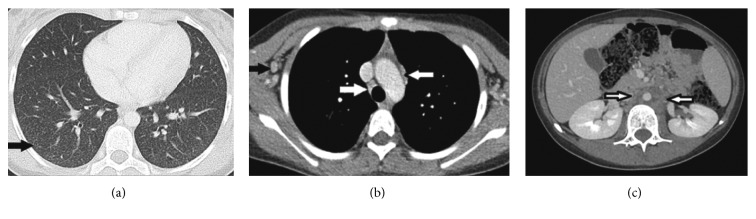
(a) Axial high-resolution chest computed tomography (CT) image through the lung bases shows multiple small (<3 mm) pulmonary nodules in a miliary pattern throughout both lungs with greatest concentration in the right lower lobe (black arrow). (b) Axial contrast-enhanced chest CT image through the superior mediastinum shows right axillary adenopathy (black arrow), right paratracheal adenopathy (white arrow), and prevascular space adenopathy (black-rimmed white arrow). (c) Axial contrast-enhanced CT image through the midabdomen shows low-density retroperitoneal adenopathy surrounding the descending thoracic aorta (black-rimmed white arrows).

Computed tomography scan demonstrated innumerable small nodules throughout the bilateral lungs and lymphadenopathy in the cervical, axillary, hilar, mediastinal, and retroperitoneal regions. The largest nodal conglomerate in the retroperitoneum measured 4.4 cm (Figure 2). Pathology of a bone marrow biopsy demonstrated hypocellularity and noncaseating granulomas. Lymph node biopsy revealed plasmacytosis and increased histiocytes with no evidence of malignancy. Acid-fast bacillus, Fite, and methenamine silver stains were negative in both tissues. Bronchoscopy revealed no significant airway edema or erythema, and no secretions. Evaluation for tuberculosis (TB) and non-TB mycobacterial infections was negative, including QuantiFERON-TB Gold, mycobacterial cultures, and/or TB PCR from sputum, bronchoalveolar lavage (BAL) fluid, lymph node tissue, blood, stool, and/or CSF. Serum antibodies to *Toxoplasma gondii*, *Treponema pallidum, Brucella* species, *Coccidioides immitis*, and *Histoplasma capsulatum* were negative. However, urine and serum *H. capsulatum* antigens were positive with values greater than the upper limits of quantification. Ultimately, *H. capsulatum* grew in fungal cultures from blood, BAL fluid, and lymph node tissue.

### 2.2. Outcome

Treatment was initiated with liposomal amphotericin B (5 mg/kg/day) on DOI 24. ART consisting of dolutegravir and emtricitabine-tenofovir alafenamide was begun on DOI 26. The patient began to feel subjectively better and appetite improved, but spiking fevers up to 42°C persisted until DOI 37. Hospital course was complicated by pancytopenia thought to be secondary to disseminated fungal infection, acute kidney injury attributed to liposomal amphotericin B, and liver enzyme elevation that was likely multifactorial. Per hospital protocol, the acute kidney injury was monitored with daily weights, serum creatinine measurements, and strict documentation of intakes and outputs. Oral itraconazole (10 mg/kg/day) was initiated on DOI 36 given improving fevers and decreasing CRP and serum *H. capsulatum* antigen. Liposomal amphotericin B was discontinued on DOI 46. Throughout the course of treatment, serum itraconazole and dolutegravir were monitored to ensure therapeutic levels were obtained. At discharge on DOI 47, the patient's CD4 T-cell count had improved to 215 cells/mm^3^ and HIV RNA PCR had decreased to 127 copies/mL.

Discussion with the patient's mother revealed that she was also living with HIV and was adherent to ART with an undetectable viral load. The mother reported receiving prenatal care during her pregnancy but was unsure whether she was tested for HIV. The patient was born via cesarean section due to eclampsia and was not breastfed. The mother was diagnosed with HIV when the patient was 4 years old. The mother reported that the patient had not been exposed to her blood and had never received a blood transfusion. She also denied any suspicion of sexual abuse. An investigation by the Colorado Department of Public Health and Environment determined that vertical transmission was the most likely source of infection. During the hospital stay, a multidisciplinary team collaborated with the patient's mother to disclose the diagnosis to the patient and provide support.

## 3. Discussion

This case highlights the need to consider HIV in the differential diagnosis of fever without source, even in previously healthy children without obvious risk factors. This patient likely acquired HIV perinatally, which makes this one of the latest presentations of vertically transmitted HIV reported in the literature [4]. Untreated HIV-infected infants are at high risk of rapid disease progression and death in the first year of life [5]. Infants who survive beyond the first year generally experience less rapid disease progression, although nearly 40% progress to AIDS or death by age 6 [6]. This patient's severe illnesses in the first 7 years of life, including bronchiolitis requiring hospital admission and varicella requiring nasogastric nutrition, may have been early clues of underlying immunodeficiency. Of note, despite the finding of detectable HIV in this patient's CSF, she did not present with any neurocognitive delay or signs of encephalopathy.

This patient was not diagnosed with HIV until 11 years after she was believed to have acquired the infection. So-called “late presenters” often have severe immunodeficiency and/or an AIDS-defining illness at the time of diagnosis. Late presenters are at risk for treatment failure, disease progression, and increased mortality [7]. Within the U.S., immigrants are more likely to be late presenters, potentially due to a variety of factors including limited healthcare access, language barriers, financial insecurity, or decreased understanding of HIV [7, 8]. Perhaps, the most important contributor to late diagnosis of HIV is low perception of HIV risk among both patients and providers, underscoring the need to consider this diagnosis even when it seems unlikely [7, 9].

This patient presented with progressive disseminated histoplasmosis (PDH) as her AIDS-defining illness. PDH is a rare opportunistic infection in children; invasive bacterial infections, herpes zoster, esophageal candidiasis, and infections caused by *Mycobacterium avium* complex or *Pneumocystis jirovecii* occur much more frequently [10]. This case is also notable because *H. capsulatum* is not endemic to the region where this patient presented, although it is endemic to much of Central America and South America. Among people living with HIV in these regions, the incidence of *H. capsulatum* and associated mortality is similar to that of TB [11]. We suspect that this patient initially acquired *H. capsulatum* in her country of origin and did not manifest signs of disseminated disease until her immune status deteriorated and the fungus reactivated. Indeed, cases of PDH have been reported in HIV-infected children who immigrated to nonendemic regions of the U.S. from Central America or South America [12]. Although PDH is rare in nonendemic areas, this case demonstrates why it is critical to keep a broad differential diagnosis for opportunistic infections in immunocompromised patients, especially if they have lived in or traveled to countries where particular pathogens may be more pervasive.

Multiple diagnostic tests are available for *H. capsulatum*, including histopathology; fungal culture of blood, bodily fluids, or tissues; antigen detection in urine, serum, or other bodily fluids; and serologic testing. Serologic testing is readily available and frequently used as a screening tool in the U.S., but results can be falsely negative, especially in immunocompromised hosts [13]. The sensitivity of serologic testing in immunocompromised hosts with PDH may be as low as 71% compared to 95–100% in immunocompetent hosts [14]. Antigen testing is most useful in patients with a significant fungal burden, but there can be cross-reactivity with other dimorphic fungi, including blastomycosis and paracoccidioidomycosis. Antigen testing is also useful for monitoring the response to treatment, as levels should decline with effective therapy and rise with disease relapse [13]. *H. capsulatum* antigen testing allowed us to diagnose this patient's PDH and initiate appropriate therapy more than a week before the first fungal culture result became available. This patient responded rapidly to the initiation of liposomal amphotericin B and ART, with clinical improvement as well as falling serum *H. capsulatum* antigen and rising CD4 counts. Of note, the immune reconstitution syndrome rarely occurs in HIV-infected adults with PDH and has never been described in children with PDH, which is consistent with this patient's course [15].

In conclusion, we describe the late diagnosis of HIV in an 11-year-old girl presumed to have acquired the infection at birth, who presented with PDH as her AIDS-defining illness. This case demonstrates the importance of screening for HIV in pediatric patients and of considering each patient's exposure history carefully when developing a differential diagnosis for prolonged fever. This case also emphasizes the importance of choosing the most useful diagnostic test in immunocompromised patients.

## References

[1] Newell M.-L., Coovadia H., Cortina-Borja M., Rollins N., Gaillard P., Dabis F. (2004). Mortality of infected and uninfected infants born to HIV-infected mothers in Africa: a pooled analysis. *The Lancet*.

[2] Spira R., Lepage P., Msellati P. (1999). Natural history of human immunodeficiency virus type 1 infection in children: a five-year prospective study in Rwanda. *Pediatrics*.

[3] Krasinski K., Borkowsky W., Holzman R. S. (1989). Prognosis of human immunodeficiency virus infection in children and adolescents. *The Pediatric Infectious Disease Journal*.

[4] Wei H.-H., Tsai L.-P., Wu P.-S. (2016). Adolescent onset of vertically transmitted untreated AIDS: a report of one case. *Tzu Chi Medical Journal*.

[5] Violari A., Cotton M. F., Gibb D. M. (2008). Early antiretroviral therapy and mortality among HIV-infected infants. *New England Journal of Medicine*.

[6] Blanche S., Newell M.-L., Mayaux M.-J. (1997). Morbidity and mortality in European children vertically infected by HIV-1. *Journal of Acquired Immune Deficiency Syndromes and Human Retrovirology*.

[7] D’Arminio Monforte A., Antinori A., Girardi E. (2012). HIV-infected late presenter patients. *AIDS Research and Treatment*.

[8] Saganic L., Carr J., Solorio R., Courogen M., Jaenicke T., Duerr A. (2012). Comparing measures of late HIV diagnosis in Washington state. *AIDS Research and Treatment*.

[9] Schwartz S. L., Block R. G., Schafer S. D. (2014). Oregon patients with HIV infection who experience delayed diagnosis. *AIDS Care*.

[10] Dankner W. M., Lindsey J. C., Levin M. J. (2001). CD4 Correlates of opportunistic infections in children infected with the human immunodeficiency virus managed before highly active antiretroviral therapy. *The Pediatric Infectious Disease Journal*.

[11] Adenis A. A., Valdes A., Cropet C. (2018). Burden of HIV-associated histoplasmosis compared with tuberculosis in Latin America: a modelling study. *The Lancet Infectious Diseases*.

[12] Saidinejad M., Burns M. M., Harper M. B. (2004). Disseminated histoplasmosis in a nonendemic area. *The Pediatric Infectious Disease Journal*.

[13] Wheat L. J. (2006). Histoplasmosis: a review for clinicians from non-endemic areas. *Mycoses*.

[14] Guimarães A. J., Nosanchuk J. D., Zancopé-Oliveira R. M. (2006). Diagnosis of histoplasmosis. *Brazilian Journal of Microbiology*.

[15] Nacher M., Sarazin F. D. R., El Guedj M. (2006). Increased incidence of disseminated histoplasmosis following highly active antiretroviral therapy initiation. *JAIDS Journal of Acquired Immune Deficiency Syndromes*.

